# Multiscale Bowel Sound Event Spotting in Highly Imbalanced Wearable Monitoring Data: Algorithm Development and Validation Study

**DOI:** 10.2196/51118

**Published:** 2024-07-10

**Authors:** Annalisa Baronetto, Luisa Graf, Sarah Fischer, Markus F Neurath, Oliver Amft

**Affiliations:** 1 Hahn-Schickard Freiburg Germany; 2 Intelligent Embedded Systems Lab University of Freiburg Freiburg Germany; 3 Chair of Digital Health Friedrich-Alexander Universität Erlangen-Nürnberg Erlangen Germany; 4 Medical Clinic 1 University Hospital Erlangen Friedrich-Alexander Universität Erlangen-Nürnberg Erlangen Germany; 5 Deutsches Zentrum Immuntherapie Erlangen Germany

**Keywords:** bowel sound, deep learning, event spotting, wearable sensors

## Abstract

**Background:**

Abdominal auscultation (i.e., listening to bowel sounds (BSs)) can be used to analyze digestion. An automated retrieval of BS would be beneficial to assess gastrointestinal disorders noninvasively.

**Objective:**

This study aims to develop a multiscale spotting model to detect BSs in continuous audio data from a wearable monitoring system.

**Methods:**

We designed a spotting model based on the Efficient-U-Net (EffUNet) architecture to analyze 10-second audio segments at a time and spot BSs with a temporal resolution of 25 ms. Evaluation data were collected across different digestive phases from 18 healthy participants and 9 patients with inflammatory bowel disease (IBD). Audio data were recorded in a daytime setting with a smart T-Shirt that embeds digital microphones. The data set was annotated by independent raters with substantial agreement (Cohen κ between 0.70 and 0.75), resulting in 136 hours of labeled data. In total, 11,482 BSs were analyzed, with a BS duration ranging between 18 ms and 6.3 seconds. The share of BSs in the data set (BS ratio) was 0.0089. We analyzed the performance depending on noise level, BS duration, and BS event rate. We also report spotting timing errors.

**Results:**

Leave-one-participant-out cross-validation of BS event spotting yielded a median *F*_1_-score of 0.73 for both healthy volunteers and patients with IBD. EffUNet detected BSs under different noise conditions with 0.73 recall and 0.72 precision. In particular, for a signal-to-noise ratio over 4 dB, more than 83% of BSs were recognized, with precision of 0.77 or more. EffUNet recall dropped below 0.60 for BS duration of 1.5 seconds or less. At a BS ratio greater than 0.05, the precision of our model was over 0.83. For both healthy participants and patients with IBD, insertion and deletion timing errors were the largest, with a total of 15.54 minutes of insertion errors and 13.08 minutes of deletion errors over the total audio data set. On our data set, EffUNet outperformed existing BS spotting models that provide similar temporal resolution.

**Conclusions:**

The EffUNet spotter is robust against background noise and can retrieve BSs with varying duration. EffUNet outperforms previous BS detection approaches in unmodified audio data, containing highly sparse BS events.

## Introduction

There are various diagnostic tools to assess bowel motility, including questionnaires, ultrasound, and endoscopic examinations [1,2]. However, there is a lack of computational tools to monitor digestion continuously across the gastrointestinal tract. Abdominal examinations using a stethoscope (ie, auscultation of the bowel) is a common clinical practice to interpret bowel sounds (BSs) [3]. While BSs could help examiners perform diagnoses [4,5], auscultation is mostly done for a few minutes only [6]. However, BSs occur sparsely over time, have varying patterns, and often exhibit low volume. Previous investigations (eg, [7]) recommended recording BSs with multiple sensors and over longer periods to maximize the amount of BS observations. Craine et al [8] reported that changes in BS occurrences across different digestive phases were statistically different in patients with irritable bowel syndrome and Crohn disease (CD). Later studies (eg, [9]) showed that digestion analysis based on BSs could support bowel motility assessment as well as monitoring food intake. For instance, an increased number of BS events could indicate bowel hyperactivity, caused by, for example, gastroenteritis or inflammatory bowel disease (IBD) [6]. Yao and Tai [10] recorded BSs across patients with CD, patients with ulcerative colitis (UC), and healthy controls. The authors reported that patients with CD showed the highest BS peak frequency, while patients with UC had the highest BS event count per unit time. Consequently, spotting BS occurrences in continuous audio could provide important information to assess digestion. To date, however, the clinical assessment based on BS remains qualitative and lacks quantification of BS characteristics [11]. For all of the aforementioned applications, short manual auscultation is considered challenging, as it provides examiners with insufficient information on dynamic bowel conditions.

Various wearable prototypes were proposed to record BSs in healthy volunteers and patients with digestive disorders (eg, [12,13]). Study protocols were primarily designed to observe BSs under controlled laboratory settings, that is, while participants laid down and rested, to minimize noise artifacts. The large amount of audio data that could be recorded by wearable systems renders a manual analysis infeasible.

Previous studies (eg, [14]) have attempted to reduce the amount of audio data to be manually analyzed with segment-based approaches that detected audio sections containing BS events. Moreover, methods were proposed to improve BS event detection and ease expert examination, by determining the onset and offset of the BS patterns in audio data streams (eg, [9,15]). Nevertheless, most algorithms were tested on balanced data sets or selected subsets of the recordings only, from dozens of minutes to a few hours. However, when collecting data with a wearable device, the BS ratio of relevant events, for example, BSs versus other surrounding sounds, largely influences retrieval performance, which reflects a basic problem in pattern spotting [16]. Specifically, in naturalistic, unmodified audio data, BS events appear sparsely and their low amplitude compared with other body sounds, for example, lung sounds, hampers BS spotting. For example, Ficek et al [15] reported that temporal sparsity of BSs could increase the false-positive rate. Previous studies have shown that BSs can vary in duration, from dozens of milliseconds to a few seconds [17,18]. Hence, the key challenge is to spot BS events, embedded in a large amount of irrelevant audio data, commonly referred to as the NULL class. To spot very short BS events (ie, those <100 ms), detection algorithms need to maximize temporal resolution, which is usually done by minimizing the sliding window size used to inspect the data stream. However, reducing the sliding window size removes context from the audio data, and thus may not improve recognition performance for BSs far shorter than 1 second.

In this paper, we present a BS spotting method based on a deep neural network (DNN) model. Our DNN model spots BS events by analyzing a continuous data stream recorded with a wearable device at 10-second audio segments. Using a multiscale approach, we can retrieve BS event onset and offset at a temporal resolution, that is, the smallest prediction duration, of 25 ms. Our approach is inspired by the way humans perform auscultation: particularly, for BS shorter than 1 second, experts would listen to the audio data surrounding the BS event to obtain an acoustic context. We evaluated our spotting approach on continuous BS recordings collected across different digestive phases, including sedentary activities and food intake. Unlike previous studies, we tested our approach on audio data having natural BS temporal distribution, that is, no resampling was applied to our data set.

The paper provides the following contributions:

- We present a DNN-based method for BS spotting in continuous data streams. Our model achieves a temporal resolution of 25 ms through a multiscale approach.
- We evaluate our model on 136 hours of annotated audio data recorded from 18 healthy participants and 9 patients with an IBD, in total including more than 11,000 annotated BS events. To spot BS events, we do not discern between healthy controls and patients with IBD, but focus on common BS acoustic properties across the different bowel conditions.
- We analyze spotting errors over the unmodified audio data streams. In addition, we analyze our model’s performance under various signal-to-noise ratios (SNRs), for different BS event durations, and by varying the temporal sparsity of BS events (ie, BS ratio).

## Methods

### Overview

Here, we describe the DNN model proposed for BS detection. Subsequently, we detail the spotting procedure, the BS evaluation study, and our evaluation methods.

### Efficient-U-Net Model

**Figure 1:**
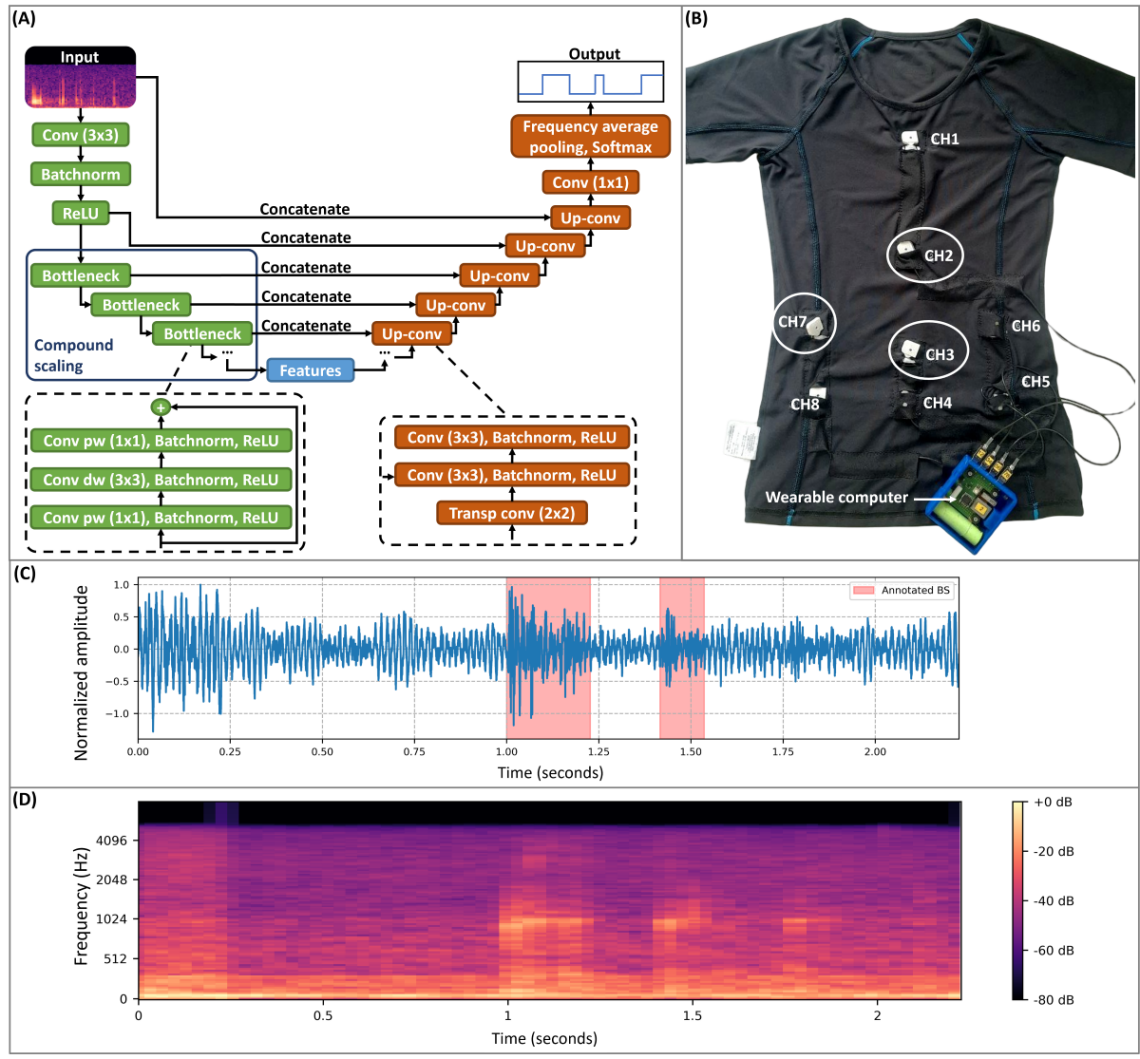
(A) Architecture of the proposed Efficient-U-Net (EffUNet) for bowel sound (BS) spotting. The model took an audio spectrogram as an input and extracted relevant features with EfficientNet-B2 during the encoding (green boxes). Subsequently, features were decoded, that is, upsampled and concatenated with higher-resolution features to locate them on the original spectrogram (orange boxes). Finally, the obtained 2D features were converted to a BS detection mask by applying average pooling along the frequency dimension and a Softmax operation to the obtained 1D temporal maps. Spectrogram frames with highest BS class probability were identified as containing BS. (B) Inside out of the smart T-shirt showing the embedded electronics. Microphones and the wearable computer were protected and isolated by 3D printed covers. Microphones CH2, CH3, and CH7 (white circles) were used during BS annotation. (C and D) Illustration and time-frequency representation of 2 expert-annotated BS events in the continuous data stream collected from 1 study participant with very different BS event duration. batchnorm: batch normalization; Conv: convolution; dw: depthwise; pw: pointwise; ReLU: rectified linear unit; transp: transposed; up-conv: transposed convolution.

Figure 1 illustrates the Efficient-U-Net (EffUNet) DNN model architecture. The proposed model was based on UNet [19] and EfficientNet [20] models, hence the name EffUNet. In total, EffUNet has approximately 18.1 million parameters.

UNet is a convolutional neural network (CNN) that was originally proposed for biomedical image segmentation [19]. The model name is given by its U-shaped architecture, which is composed of an encoder followed by a decoder network. The encoder extracts relevant features from the DNN input, and the decoder generates a segmentation mask by upsampling features from the encoder’s last layer and concatenating them with higher-resolution features extracted from the encoder’s earlier layers. Each block of the decoder is therefore composed of a 2×2 transposed convolution (up-conv), followed by two 3×3 convolutions. Upsampling restores the original input resolution, and the concatenation improves the localization of the extracted features. A final convolutional layer classifies each input point, for example, each time-frequency bin of audio spectrogram *F_k_*, with 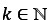. We based our approach on the UNet architecture because of its high classification resolution compared with the input data size (ie, it could classify images by pixels). Other common CNN architectures (eg, [21]) usually provide 1 prediction per input data, for example, predict object presence in an image, thus omitting other relevant information, such as the object location in the image. In audio processing, architectures similar to UNet have been used mainly for source-separation tasks [22].

In computer vision segmentation tasks, the model output is usually a 2D map with the same dimensions as the input data. In our work, EffUNet takes an audio spectrogram as an input and returns a binary detection mask in the time domain. To obtain a 1D mask, we applied an average pooling along the frequency dimension and a Softmax function to the model output. EffUNet thus classified each spectrogram time bin *F_k_* as containing either BS or non-BSs (NBSs). Thus, the spotting temporal resolution corresponds to the audio spectrogram frame length.

As an encoder, several CNN architectures were used and tested in computer vision to improve model performance (eg, residual network [21]). In our work, we used EfficientNet [20] as an encoding model. Similar architectures based on combined EfficientNet and UNet were already proposed in computer vision tasks with promising results (eg, [23]). EfficientNet architectures were introduced to improve image classification performance while reducing the amount of model parameters. The simpler architecture compared with other common CNNs makes EfficientNet suitable for mobile and edge computing applications. In EfficientNet, convolution operations are performed by a bottleneck block: (1) an inverted bottleneck (1×1) convolution, (2) a depthwise (3×3) convolution to extract features, and (3) a pointwise (1×1) convolution to linearly combine the features. Similarly, to standard convolutional layers, a batch normalization layer and a linear layer with rectified linear unit activation are applied after each convolution. In addition, residual connections are added between bottleneck blocks. Different EfficientNet configurations are available with compound scaling, that is, simultaneous increase of features count, number of layers, and input data resolution. We selected the EfficientNet-B2 configuration for our detection model. The EfficientNet-B2 architecture was already used in audio tagging tasks with promising performance [24].

### EffUNet Spotting Procedure

#### Data Preprocessing and Training Pipeline

From audio data, log-Mel spectrograms were extracted to train and evaluate EffUNet. Here, we detail the data preprocessing and training pipeline, including transfer learning and data augmentation. Subsequently, we describe the spotting implementation.

#### Audio Preprocessing

Recordings were filtered with a high-pass biquadratic filter (cutoff: 60 Hz) to remove signal offset. Subsequently, recordings were split into nonoverlapping audio segments *S_i_* with duration *δ*=10 seconds. Each audio channel was preprocessed for BS spotting independently. We defined each audio segment *S_i_* as a set of samples:

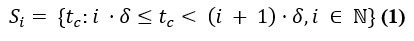



where 
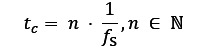
 is a time series sample.

Each audio segment was converted to a log-Mel spectrogram using 128 frequency bins, a sliding 25-ms window, and a stride length of 10 ms. As described in the “Efficient-U-Net Model” section, the 25-ms window corresponds to the spotting temporal resolution of EffUNet. Hanning windowing was applied to the sliding windows. Each resulting spectrogram had 128 Mel bins and 998 frames. According to EfficientNet-B2 pretraining [24], we zero-padded the spectrogram along the time axis to obtain 1056 time bins. The obtained spectrograms were standardized.

For every *S_i_*, we defined the audio spectrogram time bins *F_k_* as follows:

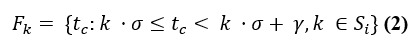



where 
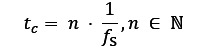
 is a time series sample. The sliding window duration γ=25 ms·*f_S_* and the stride length σ=10 ms·*f_S_* in all time series samples.

Every annotated BS event *e_j_* can be denoted as a set of time series samples as follows:

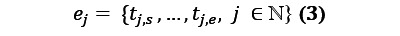



where *t_j,s_* and *t_j,e_* are the onset and offset of BS event *e_j_* in time series samples, respectively.

For model learning, BS manual annotations were converted to audio spectrogram ground truth masks according to the approach outlined by Ficek et al [15]: a spectrogram frame *F_k_* was defined as containing BSs (*F_k_*_,BS_) if the time overlaps 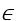 between the spectrogram frame and a BS event *e_j_* was ≥50%. Thus, for set *F_k_*_,BS_:

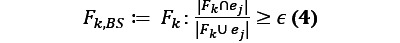



where 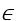
=0.5 is the temporal overlap and | · | is the set cardinality. Otherwise, the spectrogram frame was denoted as containing NBSs (*F_k_*_,NBS_), that is, the NULL class. We define supersets of all spectrogram frames as *F*_*k,*BS_
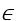
*F*_BS_ and *F*_*k,*NBS_
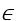
*F*_NBS_. Therefore, for each audio segment *S_i_,* we obtained from EffUNet a binary mask *M_i_* denoted as *F_k_*. As with the log-Mel spectrograms, we zero-padded the binary masks *M_i_* along the time axis to obtain 1×1056 time bin masks.

#### Transfer Learning

The EffUNet encoder, that is, EfficientNet-B2, was initialized with pretraining weights from AudioSet [25]. AudioSet is to date the largest audio data set, containing over 500 audio classes with over 2 million 10-s audio clips (ie, >5000 hours of audio data). AudioSet contains sound examples from daily living, including, among others, speech, environmental sounds, and BSs. We believe that pretraining on a large variety of sounds could improve the spotting robustness against background noise and other artifacts. As both AudioSet and the BS recordings of our study were sampled at the same frequency (ie, 16 kHz), the pretrained encoder feature extraction was compatible with our BS data. Nevertheless, because no onset and offset of audio events were originally provided in AudioSet, no pretraining could be applied to our EffUNet decoder. Therefore, the decoder weights were initialized with He initialization [26].

#### Training and Data Augmentation

EffUNet training parameters were selected according to the Pretraining, Sampling, Labeling, and Aggregation pipeline [24]. After model initialization, EffUNet was trained for 25 epochs using an imbalanced batch size of 32 and an initial learning rate of 1 × 10^−4^. The learning rate was subsequently reduced with a decay of 0.85 for each epoch, starting from the sixth epoch. Adam optimizer [27] was used with weight decay of 5 × 10^−7^, *β*_1_=0.95, *β*_2_=0.999. We used the following loss function for optimization:

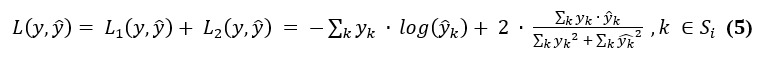



where 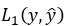 is the cross-entropy loss [28] calculated between the prediction 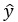 and the ground truth *y*, and 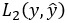 is the dice loss [29]. While the cross-entropy loss maximizes the model performance to classify single spectrogram time bins, the dice loss maximizes the similarity between the predicted binary mask and the ground truth (ie, expert BS annotation).

During the training, the input audio spectrograms were randomly transformed to improve the model generalization. On each batch, time-frequency masking [30] was applied to up to 24 frequency bins and up to 10% of the time bins. In addition, spectrograms were randomly shifted along the time axis with a maximum shift of +10 or –10 time bins. Random white noise with magnitude in the range [0, 0.1) was also added to the input spectrogram. For the evaluation, we selected the model weights obtained at the end of the training.

#### Spotting Implementation

The binary masks *M_i_* obtained from EffUNet were converted to onset/offset predictions of BS events. Spotted BS events 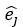 were described as follows:

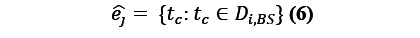

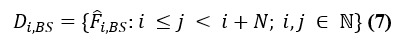





where 
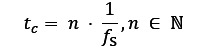
 is a time series sample, and *D_i_*_,BS_ is the set of *N* consecutive overlapping audio spectrogram time bins 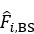 that were detected as containing BSs.

### Evaluation Study and Data Preprocessing

#### Study Protocol

The study involved 27 participants (13 females, aged 21-69 years; clothing sizes: S-XL; and BMI 17.2-32.2 kg/m^2^). Among the 27 individuals, 9 were patients with IBD. Table 1 illustrates the population characteristics of our data set. After signing written consent, participants were invited to the laboratory in the morning before breakfast.

**Table 1:** Characteristics of the population included in this study.

Cohort			IBD^a^		UC^b^		CD^c^		IBD activity		IBD remission		Healthy		Total
Participants, n			9		6		3		6		3		18		27
**Sex, n**															
	Male	3		1		2		5		2		11		14	
	Female	6		5		1		1		1		7		13	
Age (years), median (range)			39 (23-69)		36 (23-69)		39 (39-47)		33 (23-69)		47 (45-58)		28 (21-56)		28 (21-69)
BMI (kg/m^2^), median (range)			24.6 (17.9-26.2)		22.8 (17.9-26.0)		24.6 (24.2-26.1)		24.4 (17.9-25.3)		26.0 (18.4-26.1)		22.3 (17.1-32.2)		22.5 (17.2-32.2)

^a^IBD: inflammatory bowel disease.

^b^UC: ulcerative colitis.

^c^CD: Crohn disease.

A smart T-shirt (GastroDigitalShirt) [31] was used to record BSs from 8 embedded digital miniature microphones (SPH0645LM4H-B; Knowles) aligned on the abdomen. Microphones were positioned according to the 9-quadrant reference abdominal map and arranged to follow the digestive process. For example, the first channel was placed on the esophagus, the second channel on the stomach. A belt-worn computer collected and saved all microphone channels at *fs*=16 kHz. A tight-fitting design and various sizes were used to ensure comfort and optimal skin attachment. The fabric was based on elastane, thus highly stretchable. The cloth cut was based on a compression T-shirt to minimize noise artifacts as a result of motion. Different cloth cuts for females and males were prepared to fit all body shapes and provide optimal comfort. Figure 1 shows our wearable prototype and the embedded electronics.

Participants were asked to put on the smart T-shirt and audio was continuously recorded from 1 hour before breakfast (fasting phase) to 1 hour after breakfast (postprandial phase). To avoid abnormal bowel motility stimulation, induced by, for example, physical movements [32], participants laid down and quietly relaxed when there was no meal intake or other activity. They were recommended to read a book, watch or listen to multimedia on a tablet, or sleep. Although participants were relaxing, they could move on the lounge chair, if desired. Moreover, participants were required to stand up and sit down as per the study protocol, so motion artifacts could be included in the recording. While eating breakfast, participants sat at a table and were allowed to talk to the study personnel or move freely around the room. The audio was continuously recorded during the whole session. Participants were allowed to drink water throughout the recording and could pause it anytime for a break (eg, to visit the toilet). Along with BSs, other sound events could be captured (ie, NULL class data). For instance, conversations between participants and study personnel and other environmental sounds from the room surroundings, for example, traffic as well as activities and voices outside the recording room, could be recorded. In addition, voluntary body position adjustments and eating or drinking could introduce noise artifacts.

Upon completing the recording protocol, study participants were asked to rate the T-shirt’s comfort and usability to confirm that it could be worn for the recording duration. The assessment was based on the wearable comfort assessment questionnaire [33]. The study participants reported no discomfort caused by the embedded electronics.

#### BS Annotation

Recordings were annotated by pairs of raters through audio and visual inspection of the raw audio data using Audacity (The Audacity Team). Annotations were sample specific, that is, no quantization of the BS events’ onset/offset was performed. An example of annotated BS events with different durations is presented in Figure 1. For raters to identify BS events with varying durations and amplitudes in the recordings, view time resolution in Audacity had to be adjusted. On average, each rater required 8-12 hours to label 1 hour of recording, depending on event rate (ie, the number of BSs per unit time) and noise level. As a result of the time-consuming annotation process, only a subset of the recordings was annotated by more than 1 rater to evaluate interrater agreement (see below). The remaining data were labeled by 1 of the raters and the annotations were checked by the other rater. Among all participants, audio data from the sensors positioned on the stomach (CH2) and the small intestine above the navel (CH3) were annotated (see Figure 1 for a sensor map). As IBD usually affects the distal part of the gastrointestinal tract, in the patient group and in some individuals from the healthy group, an additional microphone placed on the distal part of the large intestine (CH7) was included in the annotation to evaluate our spotting approach with additional BS patterns. Because of SNR limitations, the channel located at the large intestine could not be annotated for all participants. The annotation was performed on each recording channel separately because BSs could be recorded at 1 or more locations depending on the sound propagation across the abdomen. The position of annotated audio channels on the T-shirt is shown in Figure 1.

Based on BS features reported in the literature [17,18], as well as preliminary auscultation sessions, and early annotation reviews, labeling guidelines were selected and agreed upon between raters: BS duration had to be 18 ms or more, and consecutive BS events with sound-to-sound interval less than 100 ms were marked as a single event. Noisy or BSs not visible in the audio signal were labeled as tentative.

**Figure 2:**
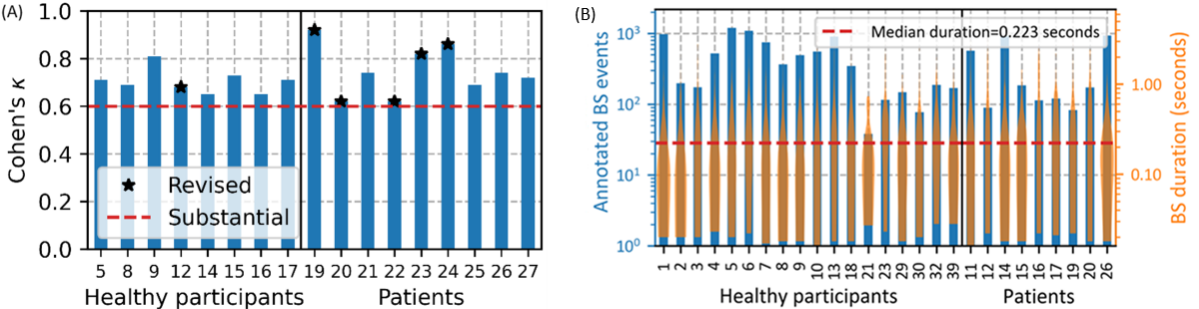
(A) Interrater agreement per study group. The evaluation was performed on a subset of the study participants. Overall, the agreement on the nontentative bowel sound (BS) annotations was substantial. (B) Amount and duration distribution of BS annotated per participant. Most BSs are short (median duration 223 ms).

Cohen κ interrater agreement was used to evaluate the annotation quality. Two raters labeled the first 30 minutes of recordings from 8 healthy participants and 9 patients. After the agreement evaluation for each participant’s recording, a label review session was conducted to discuss and revise any BSs with disagreement. If an agreement of κ<0.6, indicating slight to moderate disagreement, was observed for a participant data set, then the agreement score was recalculated based on an additional 10 minutes of the participant’s recording after the review and revision. Overall, in the healthy group and the patient group, agreement on nontentative BS annotations was substantial, with Cohen κ of 0.70 and 0.75, respectively. As the data imbalance between BSs and NBSs increases, the maximum achievable agreement between raters decreases. Therefore, agreements are deemed fair to good beyond chance for scores between 0.40 and 0.75 [34]. Figure 2 illustrates the interrater agreement per study group.

Overall, 11,482 BSs plus 3801 tentative BSs were annotated on approximately 136 hours of audio. The annotated BSs had a total duration of 1.22 hours, with 52.39 minutes recorded from the healthy group and 20.71 minutes recorded from the patient group. Of the total nontentative annotated BSs, 3215 were observed at the stomach, 5667 at the small intestine, and 2600 at the large intestine. Because of the noisy signal patterns that could affect the spotting performance, tentative BSs were not included in the analysis and, therefore, were considered NBSs (ie, belonging to the NULL class). The quantity and duration of annotated BSs across all participants are shown in Figure 2. As reported by previous studies [17,18], BS event duration ranges from 18 ms to a few seconds. However, most annotated BSs have a very short duration (<500 ms).

### Evaluation Methods

#### Validation Method

Leave-one-participant-out (LOPO) cross-validation (CV) was used to evaluate spotting performance: audio data from all but 1 participant were used to train the DNN, and its performance was evaluated on the excluded participant’s data. Performance statistics were obtained from the results of each validation set.

#### Evaluation Metrics

Precision and recall (PR) metrics and *F*_1_-score were used to evaluate spotting performance across all testing data. Metrics were calculated using a samplewise approach based on Mesaros et al [35], that is, model predictions and BS annotations were compared sample-by-sample: *t_i,_*_S_=1 (*f_S_*≈0.06 ms). Thus, our evaluation approach was independent of the spotting algorithm resolution. Furthermore, we compared directly with BS annotations without considering their spotting frame–adjusted versions.

We analyzed spotting detection errors with the 2-class segment error metric [36]. False-positive FP*_i_* were marked as merge errors if they connected 2 consecutive events *e_j_*, overfill errors if FP*_i_* occurred at the beginning or end of an event *e_j_*, and insertion errors otherwise. False-negative FN*_i_* were marked as fragmentation errors if FN*_i_* occurred within 1 event *e_j_*, underfill errors if they occurred at the beginning or end of an event *e_j_*, and deletion errors otherwise. For each LOPO fold, we derived the overall detection timing errors as time duration.

Model performance statistics were described using median and IQR values. IQR was determined as the difference between quartile Q1 (ie, the mid value between the median and the minimum) and quartile Q3 (ie, the mid value between the maximum and the median). We further evaluated spotting performance by analyzing PR metrics over SNR as follows:

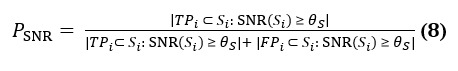

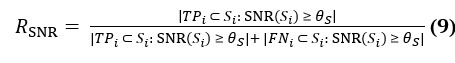





where *θ_S_* is an SNR threshold applied to audio segments *S_i_*. For each *S_i_*, SNR was computed in the log-decibel scale as the ratio between the signal power of *e_j_* 
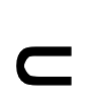
 *S_i_* and the background noise in *S_i_*.

Moreover, we analyzed PR metrics over BS duration. To estimate TP*_i_*, FP*_i_*, and FN*_i_* depending on BS duration, we only considered annotated events *e_j_* and detected event 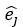 so that |*e_j_*| ≥ *θ*_D_ and 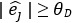, where *θ*_D_ was a BS duration threshold.

Retrieval generalization was additionally evaluated by analyzing PR over event rate. Event rate was defined as BS events per time unit according to Amft [16]. To compare our model performance with related work, we converted event rate to BS ratio (ie, the ratio between spectrogram time bins containing BSs and total time bins) as follows:

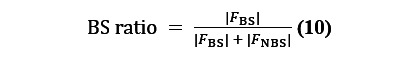



For each validation fold, we swept the BS ratio from 0.00001 to 0.60 by randomly sampling *K* from *F_k,_*_BS_ and *J* from *F_k,_*_NBS_ so that BS ratio=*K*/(*K*+*J*), thus corresponding to bootstrap samples according to the count of validation folds. For each selected BS ratio, we calculated the corresponding event rate per hour of recording. Although models were not retrained on the selected BS ratios, the analysis provides insights into the performance at different class imbalance levels. We show that spotting performance depends on the BS ratio and compared our results with published works in the literature, which mostly focused on artificially balanced data sets.

#### Comparison With Prior Work

We examined the performance of existing models for BS detection on our data set. For comparison purposes, we focused on spotting models with similar temporal resolution. Segment-based spotting approaches, such as those described by [37], were excluded from our comparison because of their distinct design scope, which does not include providing BS event onset/offset. Among recent works, the convolutional recurrent neural network (CRNN) by Ficek et al [15], the CNN by Wang et al [9], and the CNN by Kutsumi et al [38] offer the highest temporal resolution. The CRNN training pipeline, originally evaluated in a data set of 53 minutes, could not be scaled to our substantially larger data set, as the CRNN optimization did not converge on our highly imbalanced data set. The CNN by Kutsumi et al [38] could not be reimplemented as it lacked methodological information (see the “Discussion” section). We, therefore, reimplemented and trained the CNN by Wang et al [9] for 30 epochs using an initial learning rate of 0.001 and a balanced batch size of 128. Adadelta optimizer with a weight decay of 10^–7^ and a decay rate of 0.95 was used to optimize the cross-entropy loss function. Unlike our EffUNet model, the CNN takes as input a log-Mel spectrogram extracted from 60 ms nonoverlapping audio segments using a 50-ms sliding window (preprocessed with Hanning windowing) and a stride length (*σ*) of 5 ms. The CNN classified each spectrogram as either containing BSs or not. We split our data accordingly and assigned each 60-ms audio segment to either the BS or the NBS class using Equation 4. We could not follow the labeling approach proposed by Wang et al [9] because the authors manually annotated each 60-ms audio segment individually rather than the continuous data stream. The acquired audio data were preprocessed by applying a high-pass filter with a cutoff of 80 Hz. According to the authors, spectrograms were standardized and no data augmentation was used during the training. To directly compare the results with EffUNet, the CNN was evaluated using LOPO CV.

### Ethics Approval

The study was approved by the Ethics Commission of the Friedrich-Alexander Universität Erlangen-Nürnberg (protocol number 73_20 B).

## Results

### BS Ratio and *F*_1_-Scores

**Figure 3:**
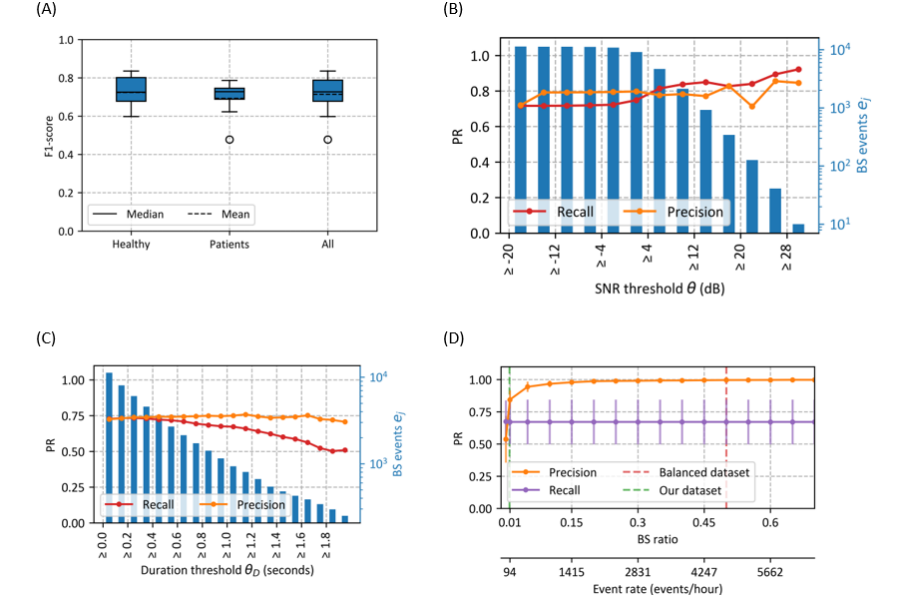
(A) Box plots of *F*_1_-scores across all participants and study groups. For both groups, a median *F*_1_-score of 0.73 was obtained; however, the patient group showed the lowest IQR. (B) Precision and recall (PR) over signal-to-noise ratio (SNR) analysis. The number of bowel sound (BS) events considered for each threshold is also shown. When the SNR is >4 dB, more than 80% of BSs were detected by Efficient-U-Net, with a precision in the range of 77%-86%. (C) PR over BS duration analysis. The number of BS events considered for each threshold is also shown. Even when including very short BSs in the analysis, our model could detect events with nearly 75% PR. (D) PR over BS ratio. Dots and error bars show median and IQR, respectively. In our data set, the BS ratio is only 0.0089. Nevertheless, 73% of BSs were recognized with 72% precision. Most studies in the literature were performed on a balanced data set. If the BS ratio was >0.05, our model would detect BSs with precision >83%.

Based on the annotated 11,482 BS events, we obtained a BS ratio of approximately 0.0089 for the data set. Figure 3 shows *F*_1_-scores across all participants and for each study group. EffUNet achieved the largest *F*_1_-score in the healthy group. For both groups, a median *F*_1_-score of 0.73 was obtained. Although the patient group yields the lowest IQR, an outlier with an *F*_1_-score of approximately 0.50 was identified.

Table 2 shows the median PR of our spotting model for all study groups. The BS spotting achieved identical median precision scores for both the healthy and patient groups. However, BSs recorded from patients proved more challenging to detect, resulting in a lower median recall compared with the healthy group.

**Table 2:** Spotting performance for all study groups. While Efficient-U-Net shows the same median precision for both study groups, the median recall was higher for healthy individuals than for patients.

Study group	Precision, median (IQR)	Recall, median (IQR)	*F*_1_-score, median (IQR)
Healthy	0.80 (0.19)	0.75 (0.19)	0.73 (0.13)
Patients	0.80 (0.23)	0.66 (0.14)	0.73 (0.09)
All	0.80 (0.19)	0.73 (0.18)	0.73 (0.11)

**Figure 4:**
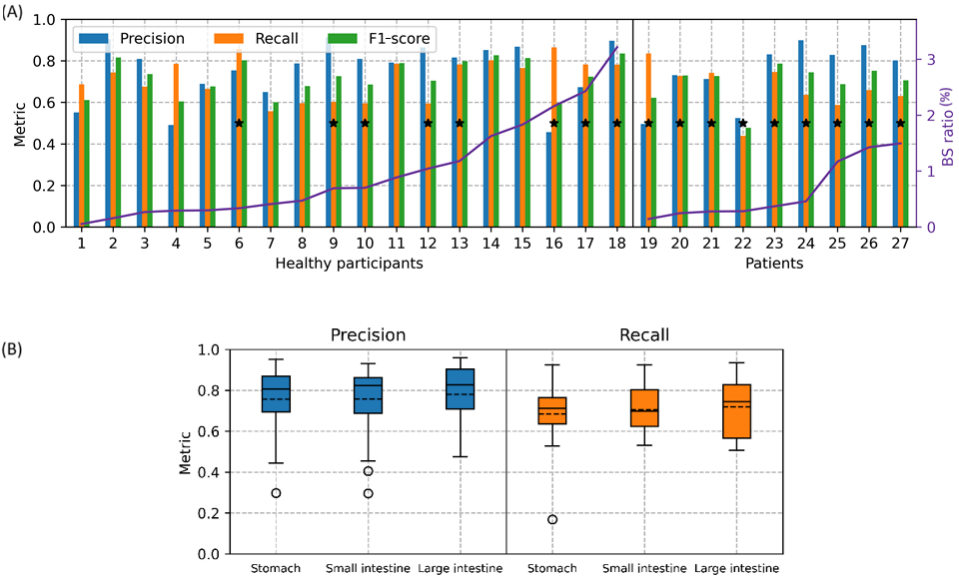
(A) Precision and recall (PR) metrics and *F*_1_-score per study participant. Bowel sound (BS) ratios per participant recording are indicated. Participants whose sensor on the large intestine was annotated are marked by an asterisk. Overall, BSs were sparser in the patient group than in the healthy group, with a peak BS ratio of 0.015 versus 0.032. The *F*_1_-score was above 60%, except in individuals in whom performance decreased due to a precision drop. (B) PR metrics comparison across the different sensor positions. For all locations, Efficient-U-Net yielded comparable median and IQR for precision. The median recall was similar for all sensor locations, while the recall IQR was largest on the large intestine.

PR metrics and *F*_1_-score per participant and BS ratio are shown in Figure 4. Overall, BSs were sparser in the patient group than in the healthy group, with a peak BS ratio of 0.015 versus 0.032. Regardless of the BS temporal distribution, EffUNet achieved an *F*_1_-score above 60%, except for an outlier in the patient group. In participants, where *F*_1_-score dropped to approximately 60%, the performance decrease was mainly a result of a drop in precision, while most BSs could be retrieved. Figure 4 also shows PR metrics per sensor location. For all locations, EffUNet yielded comparable median and IQR for precision. The median recall was similar for all sensor locations, while recall IQR was largest on the large intestine.

Timing errors using the 2-class segment error analysis across the study groups are shown in Table 3. For both healthy participants and patients, insertion and deletion timing errors were the largest, whereas fragmentation and merge errors were the lowest. Besides per-participant timing error medians and IQR, the timing error totals are shown. The total errors over the 136 hours of data were 15.54 minutes for insertions and 13.08 minutes for deletions.

**Table 3:** Spotting timing errors per participant and totals using 2-class segment error analysis. Overall, insertion and deletion errors showed the largest timing deviations for both healthy and patient groups, whereas fragmentation and merge errors showed the smallest deviations. On our data set of approximately 84 hours for the healthy group and 52 hours for the patient group, 52.4 and 20.7 minutes were annotated as bowel sounds, respectively.

Study group		Insertion	Deletion	Fragmentation	Merge	Overfill	Underfill
**Per-participant summed spotting errors (seconds), median (IQR)**							
	Healthy	15.2 (13.0)	24.0 (35.2)	1.1 (3.4)	0.5 (1.4)	4.2 (9.4)	7.2 (14.8)
	Patients	13.1 (14.1)	20.8 (57.5)	1.1 (3.3)	0.7 (0.8)	3.6 (7.4)	7.4 (21.6)
	All	14.6 (13.0)	21.9 (36.5)	1.1 (3.4)	0.5 (1.0)	4.1 (7.0)	7.4 (15.4)
**Total per-participant summed spotting errors (minutes), median**							
	Healthy	13.3	7.9	0.8	0.4	3.1	3.7
	Patients	2.3	5.2	0.3	0.1	0.9	1.8
	All	15.5	13.1	1.1	0.5	4.0	5.6

Figure 3 shows PR metrics over SNR. Annotated BS events considered within each SNR threshold θ*_S_* bin are indicated. Our model detected BSs under different noise conditions with 0.73 recall and 0.72 precision. When BSs are louder than background noise, that is, SNR>4 dB, more than 83% of BSs were recognized, with precision in the range between 77% and 86%.

PR metrics over BS duration are shown in Figure 3. Even when including very short BSs in the analysis, our EffUNet model could detect events with nearly 75% recall and precision. EffUNet recall dropped below 60% for BS duration of 1.5 seconds or more, probably because of fragmented predictions that were removed by the duration analysis procedure, that is, BS event duration below θ*_D_* (see the “Evaluation Metrics” section for details).

Figure 3 shows PR metrics over different BS ratios. At our data set’s BS ratio of 0.0089, 73% of the BSs were recognized with 72% precision. Most studies in the literature were performed on a balanced data set. If the BS ratio of our data set was over 0.05, our model would detect BSs with precision greater than 83%.

### Comparison With Prior Work

Table 4 shows a comparison of the proposed model with other methods from related work. Our approach was developed and tested on a large data set of 136 hours of recordings. Unlike other studies, the model was tested on the full, highly imbalanced data set. Despite a window of 10 seconds being fed to EffUNet during the detection, our multiscale approach can detect BSs with a temporal resolution of 25 ms. The CNN proposed by Wang et al [9] yielded a recall of 90% when tested on a balanced data set of approximately 11 minutes in total. On our substantially larger and highly imbalanced data set, however, the CNN model of Wang et al [9] only yielded a median precision of 5% (IQR 0.07) and a median recall of 74% (IQR 0.07). Optimization of the model proposed by Wang et al [9] to deal with imbalanced data may be feasible, but is beyond the scope of this work.

**Table 4:** Comparison of our model performance with other methods proposed in the literature. Stated performances were those provided by the corresponding articles.

Model	Evaluation data set size	Bowel sound ratio	Recording conditions	Temporal resolution	Precision	Recall
CRNN^a^ [15]	N/A^b^	0.0246	Nocturnal recording, clinic	10 ms	0.58	0.86
CRNN [15]	≈11 minutes	0.15	Nocturnal recording, clinic	10 ms	0.83	0.77
CNN^c^ [9]	≈15 minutes	0.50	Quiet room	60 ms	N/A	0.90
CNN [38]	2.4 hours	N/A	N/A	100 ms	0.71	0.75
LSTM^d^ [39]	≈5 hours	0.45	House rooms	1 second	≈0.94	≈0.99
Autoencoder [37]	≈81 minutes	0.50	Anechoic chamber, synthetic noise	5 seconds	0.92	0.50
Ensemble CNN [40]	49 minutes	0.50	Neonatal intensive care unit	6 seconds	0.97	0.98
CNN + Attention [14]	84 hours	0.15	Laboratory room	10 seconds	0.81	0.70
Efficient-U-Net (this work)	≈136 hours	0.0089	Laboratory/clinical room	25 ms	0.80	0.73

^a^CRNN: convolutional recurrent neural network.

^b^N/A: not applicable.

^c^CNN: convolutional neural network.

^d^LSTM: long short-term memory neural network.

## Discussion

### Principal Findings

Acoustic abdominal monitoring requires physicians to analyze BSs across different digestive phases to detect gastrointestinal disorders. Our data set comprises approximately 2 hours of continuous audio data for each of the 27 participants. We recorded various phases of digestion, from the fasting stage to the food ingestion and consequent postprandial phase. To evaluate the potential of our model in a realistic scenario, the BS natural temporal distribution was left unaltered, that is, no class resampling was applied to the data set. In addition, various activities that are typical of free living were recorded in the study, for example, eating or transition movements (ie, getting up/laying down). While participants laid in a relaxed position for part of the recording session to minimize motion-induced peristalsis stimulation [32], their actions were not constrained (eg, they could grab a bottle and drink water if desired). In particular, participants were allowed to freely move and talk during breakfast. Moreover, as the recording room was not acoustically isolated from the surroundings, various noise sources could be captured in the recording besides the artifacts introduced by the participant movements (see the “Study Protocol” section). We believe that our recording setting and data amount can be considered as a realistic representation of common activities and sedentary lifestyles. While we suggested a sedentary behavior for participants to obtain nonstimulated BS distributions (as discussed earlier), future work could evaluate BS spotting under different conditions, such as specific physical activities, sports, and stress. If necessary, these activities could be conveniently filtered out using basic detection methods, such as those based on accelerometer data.

### Spotting Temporal Resolution

BS spotting requires a temporal resolution in the millisecond scale to detect very short events (<100 ms). Previous studies have attempted to maximize the temporal resolution by minimizing the sliding window applied to inspect the audio data (eg, [9,41]). When recording BSs with wearable devices, however, the temporal sparsity of BS events could increase as a result of sensor displacements or noise artifacts. As the acoustic context decreases with sliding window duration, false-positive cases may increase, thus limiting spotting performance. Our multiscale approach can analyze continuous recordings with a temporal resolution of 25 ms while retaining 10 seconds of acoustic context in the spotting by the audio segment *S_i_.*

### Comparison Between Healthy and Patients With IBD

Our DNN model EffUNet can detect BSs with a median precision of 80% and a median recall of 73%. Although the median *F*_1_-score for healthy participants and patients was the same, BS spotting was more challenging in patients, as the difference in the median recall of 66% versus 75% indicates (Table 2). The patient group included individuals with different IBDs and varying levels of inflammation activity, which may explain the greater variability of acoustic patterns in BSs, compared with the healthy group. Our results warrant further data recordings from patients with IBD. A nested validation set could be used to analyze model hyperparameters. In this work, however, our focus was to maximize trainset size and minimize model bias. Thus, we used LOPO CV without early stopping criteria during training and other training parameters were chosen according to the Pretraining, Sampling, Labeling, and Aggregation pipeline [24]. The *F*_1_-score was above 60% for all patients, except for 1 outlier (P22; see Figures 3 and 4), where the performance of EffUNet dropped to approximately 50% as a result of the reduced recall. As the *F*_1_-score of EffUNet showed an IQR of 0.14 across all patients, we attribute the performance drop for P22 to a reduced recording quality: Less than 100 annotated BS events across all channels were documented (BS ratio=0.0028). Analysis of the false-positive rate showed that EffUNet spotted events that were marked by the raters as tentative BSs because of their noisy patterns. If tentative BSs had been included in the evaluation for P22, the model’s precision would have increased from 52% to 83%. However, tentative events were not considered in our analysis because of their noisy acoustic patterns and were labeled as NBSs. Thus, assigning tentative annotations to NBSs, that is, the NULL class, may have increased overall insertion errors and thus contributed to a conservative performance estimation. Further investigations on data collection and preprocessing, for example, adaptive noise filtering [12], could improve signal quality and consequently spotting performance as well.

### Spotting Performance Under Different Noise Conditions

Compared with other studies, where BSs were recorded using a skin-taped sensor [42], our work used garment-embedded microphones. Continuous data collection with wearable devices could further decrease the signal amplitude as a result of accidental sensor displacement and motion artifacts. Nevertheless, our approach can spot BSs with recall greater than 73% regardless of the noise level (Figure 3). In addition, our precision over SNR analysis demonstrated that our model was robust against background noise, as more than 70% of predictions corresponded to ground truth events even when SNR=–20 dB. In particular, for low SNR conditions, empirical threshold–based BS detection methods could fail, as reported, for example, by Sato et al [41]. We attribute EffUNet’s reduced number of false positives to the encoder’s pretraining on AudioSet. EfficientNet-B2 was originally trained to detect sound events in audio clips of duration δ=10 seconds [24]. In the experiments on AudioSet, EfficientNet-B2 achieved an average precision of ≈0.44 for classifying 527 sound classes. The pretraining on a large variety of noise sources could have improved the modeling of the NBS class, and thus, model precision. However, AudioSet does not provide strong audio labels (ie, event onset/offset), and therefore, no pretraining could be applied to the EffUNet decoder.

### Spotting Timing Errors

Previous studies on sound event detection (eg, [36]) highlighted that common pattern recognition evaluation metrics are insufficient to describe error types in continuous data. For instance, a model could return a fragmented prediction of a ground truth event or could recognize multiple events in 1 prediction. Previous work on BS spotting rarely analyzed detection errors besides the false-positive rate. As missed BSs will decrease the number of BS events per unit time, diagnostic approaches based on BS event count thresholding (eg, [8]) may fail to identify IBD. Furthermore, timing errors may affect the diagnosis of gastrointestinal disorders. Fragmentation or merge errors could alter natural BS acoustic characteristics (ie, spectral and temporal features), which were explored in previous studies to classify digestive dysfunctions (eg, IBD [43]) or to detect digestive events (eg, migrating motor complex [44]). Our analysis of detection errors (Table 3) showed that the performance of the EffUNet model was mainly impacted by insertions (ie, false positives) and deletions (ie, missed BSs). In 136 hours of audio data, 19.56 minutes of background noise were wrongly detected as BSs, because of either insertion or overfill errors. Insertion errors were largest in the healthy group (13.28 minutes out of 84 hours of audio data), probably because of the larger group size compared with the patient group, and consequently more variable background noise. Of the 1.22 hours of audio annotated as BSs, 18.56 minutes were not recognized because of deletion and underfill errors. Deletion errors were largest in the patient group (5.15 minutes out of the annotated 20.71 minutes), as confirmed by the lower recall compared with the healthy group (66% vs 75%). Nevertheless, our training loss (Equation 5) could minimize fragmentation and merge errors (ie, EffUNet returned prediction onset/offset according to our annotation approach). Overall, overfill and underfill errors were 4.01 and 5.56 minutes, respectively, and peaked in the healthy group. As BS annotations were converted to a binary mask to train EffUNet (Equation 4), we hypothesize that further improvements on the input data preprocessing (eg, spectrogram sliding window size γ and stride length σ) could improve the detection temporal resolution, thus minimizing overfill and underfill errors.

### Spotting Performance Over BS Event Duration

As described by previous studies [17,18], BSs present acoustic patterns of variable duration. In our study, BS length varied from 18 ms to 6.29 seconds, which created a challenging spotting task. To detect very short events (ie, <100 ms), previous work typically used a sliding window with a duration no more than the BS length (eg, [15,41]). Because of the constrained data included in the window, a spotter thus has limited information available to spot BSs. As for human experts, spotting performance could decrease when context information from the surrounding audio scene diminishes. By contrast, a larger sliding window could decrease temporal resolution, and thus yield coarse event onset/offset prediction (eg, [45]). In our work, we propose a multiscale approach: The data stream is first split into 10-second audio segments *S_i_*, and for each *S_i_*, a binary mask *M_i_* is generated. While analyzing *S_i_* with duration 𝛿=10 seconds at a time, EffUNet can detect BSs with a temporal resolution of 25 ms. Our approach could be potentially applied to other spotting tasks where events have a varying duration (eg, gesture recognition [46]). Our analysis on recall versus event duration *e_j_* showed a decrease in recall for long BSs (>1 second; Figure 3). As, in our analysis, the duration threshold *θ_D_* was applied to events *e_j_* as well as predictions 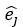, we hypothesize that predictions for longer events could have been affected by underfill or fragmentation errors and, consequently, filtered out during the analysis. Long BS events have been previously described as a sequence of single and multiple bursts interrupted by silence periods. In our BS annotation approach (see the “BS Annotation” section), silence periods between consecutive bursts of a maximum 100 ms were accepted. Therefore, some parts of a long BS (>1 second) could have been rejected by EffUNet as noise. Additional postprocessing on spotting results (eg, merging nearby detected events 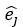) or alternative loss functions (eg, based on dice loss [29]) could improve the retrieval of long BSs (>1 second). For instance, scaling factors could be explored when combining the cross-entropy loss with the dice loss during EffUNet training. In this work, no weighting was applied when calculating the loss during backpropagation (Equation 5).

### Spotting Performance for Different Event Rates

Previous studies have already introduced shallower DNNs than EffUNet to spot BSs in the data streams, demonstrating promising results (eg, [9,15]). However, the previously reported models were trained and tested on limited, partially selected data subsets, often with a BS ratio of 0.50 (ie, class balance between BSs and NBSs; Table 4). When spotting BS events in continuous recordings that are collected in daily settings, however, a substantial BS versus NULL class imbalance must be expected (see Figures 2-4). Algorithm evaluations on a balanced data set could therefore overestimate performance for a BS ratio <<0.50. The difference can be seen between Wang et al’s [9] original report and the analysis of their CNN on our data set. However, Wang et al’s [9] CNN was designed for balanced BS detection, which limits a direct comparison with EffUNet in our study. In our data set, BSs were highly sparse, with BS ratios less than 0.035 across all participants, corresponding to event rates of approximately 100-300 events/hour. With event sparsity, the spotting challenge increases [16], especially when training and evaluating the spotter on different class imbalances. Despite the high class imbalance, however, EffUNet could retrieve BSs with a recall of 73% and a precision of 72%. By contrast, Ficek et al [15] reported a precision of 83% at a BS ratio of 0.15, but yielded a precision of 58% for a BS ratio of 0.0246. If our data set had a BS ratio of 0.15 (approximately 1400 events/hour), the precision of our DNN would reach an estimated 92% (Figure 3).

### Spotting Performance Over Sensor Location

We compared PR metrics across different sensor locations (Figure 4). EffUNet yielded comparable median recall across all locations, although recall IQR was largest at the large intestine. As performance median and IQR were comparable for all sensors, we hypothesized that the higher recall IQR could be due to abnormal BS patterns that are more likely to occur in the large intestine. Although the sensor data from the large intestine were annotated only for a subset of the study participants, performance was not affected by the data imbalance across channels.

### Limitations

While our wearable prototype design allowed us to capture BSs with multiple sensors, no sensor fusion was applied as in [12,37]. Previous experiments by Ranta et al [47] showed that the abdomen can be acoustically modeled as an absorbent material, and thus BS intensity depended on sensor distance. Our data annotation confirmed that not all BS events were captured by all channels. Given past analyses on abdominal sound propagation, we decided to minimize the model complexity and designed a single-channel spotting model. Further investigations on abdominal sound propagation may improve BS source localization and estimate the relationship between BSs and bowel movements. For instance, the EffUNet architecture could be extended to analyze multichannel recordings and locate BS sources. Preliminary studies on BS source location [47], however, suggested that further basic analyses on sound propagation in the abdominal cavity are needed. BS source localization would be beneficial for patients with IBD, for example, to locate inflammation sites noninvasively based on abnormal BS patterns and related digital biomarkers.

Although our analysis compared EffUNet spotting performance across study populations with different gastrointestinal conditions (healthy volunteers and patients with IBD), the impact of false positives on BS-based clinical gastrointestinal assessment was not evaluated. Future studies should investigate methods for digestive disorder recognition. Based on the spotting method proposed in this work, a fully automated and noninvasive approach for digestive disorder analysis may be feasible.

The usability and comfort of the wearable prototype were not analyzed in depth in this work. A full user comfort study is beyond the scope of this analysis. Nevertheless, we carefully considered user comfort during the T-shirt implementation, especially because our investigation involved patients and the longest recordings analyzed thus far. While participants did not express complaints about the wearing comfort, which confirms the efficacy of our approach, further research could explore design optimizations supported by a focused wearability assessment.

Our study encompassed 2 hours of annotated audio from 2-3 channels for each of the 27 participants, resulting in 136 hours of labeled data. To the best of our knowledge, our data set is the largest annotated BS data set reported to date. Our results justify further studies with even larger data sets. Although BSs were recorded across different digestive phases, further investigations should include data collected from extended monitoring periods, such as over multiple days, and in less constrained settings, such as in a home setting. This approach would better facilitate the correlation of physiological digestive processes with BS acoustic properties and enable the investigation of noise effects.

### Comparison With Prior Work

The goal of our work was to design and evaluate an approach to deal with imbalanced BS data gathered from body-worn microphone sensors. Therefore, when comparing EffUNet with previous studies, we focused our analysis on models with similar scope (ie, BS spotting with maximized temporal spotting resolution). As reported by Ficek et al [15], data scarcity and missing annotations as a result of labor-intensive audio recording inspection are well-known challenges in the field of BS analysis. Open BS data sets are inexistent so far, which may be due to privacy concerns associated with raw audio recordings. Moreover, BSs are often collected in different recording settings, using various wearable devices and following varying recording protocols. Consequently, benchmarking our EffUNet directly against past BS spotting models is challenging. We excluded architectures that are similar to EffUNet but were not designed for BS detection (eg, UNet [19]). According to Table 4, only 3 methods achieved spotting resolution below 100 ms. The CRNN by Ficek et al [15] could not be scaled to our much larger data set. Further, the CNN proposed by Kutsumi et al [38] could not be included in our comparison, because it lacked methodology details that are required to reimplement the model (eg, training optimizer and dropout layer parameters). Nevertheless, we reimplemented the CNN by Wang et al [9]. Compared with EffUNet, the CNN is a shallower network, with approximately 62,000 parameters versus approximately 18.1 million EffUNet. Our model outperformed the CNN by Wang et al [9] not only in temporal spotting resolution (25 ms of EffUNet vs 60 ms of the CNN), but also in spotting performance on highly imbalanced data (80% median precision of EffUNet vs 5% median precision of the CNN). Using EfficientNet for our model encoder allowed us to leverage pretraining to increase model robustness against noise, as shown in [14]. Further work may investigate whether less complex models than EffUNet could be optimized for natural, imbalanced data. Moreover, our analysis of related work (Table 4) provides a comparison of recent advances in BS spotting, showing how our work outperforms previous studies in dealing with data imbalance and temporal spotting resolution.

### Conclusions

We presented a multiscale BS spotting model based on the EffUNet architecture, to detect BSs in continuous audio data streams. AudioSet pretraining was applied to the EffUNet encoder to improve model robustness against noise. We evaluated our model using 136 hours of audio data collected from 18 healthy participants and 9 patients with IBD. Our experiments demonstrated that EffUNet can detect BSs with a median *F*_1_-score of 73% in recordings where BS events were highly sparse (BS ratio of 0.0089). With EffUNet, BSs of varying durations and under different noise conditions could be identified with a precision of 72%. Our EffUNet analysis surpassed previous approaches not only in terms of evaluation data size and temporal sparsity of BS events but also achieved one of the highest temporal resolutions. Using our approach, future analyses of BSs obtained from wearable abdominal monitoring systems could be automated without requiring manual audio data annotation.

## Abbreviations

BS: bowel sound
CNN: convolutional neural network
CRNN: convolutional recurrent neural network
CV: cross-validation
DNN: deep neural network
EffUNet: Efficient-U-Net
IBD: inflammatory bowel disease
LOPO: leave-one-participant-out
NBS: nonbowel sound
PR: precision and recall metrics
SNR: signal-to-noise ratio
up-conv: transposed convolution
